# Spontaneous pneumothorax from cryptococcal pneumonia in systemic sclerosis: a case report

**DOI:** 10.1186/1752-1947-5-309

**Published:** 2011-07-13

**Authors:** Kwanreuthai Sripavatakul, Chingching Foocharoen

**Affiliations:** 1 Department of Medicine, Faculty of Medicine, Khon Kaen University, Khon Kaen, 40002, Thailand

## Abstract

**Introduction:**

Spontaneous pneumothorax is usually found in people with systemic sclerosis who have extensive pulmonary fibrosis with enlarged sub-pleural blebs. We report a case of spontaneous pneumothorax caused by cryptococcal pneumonia in a patient with systemic sclerosis with minimal sub-pleural emphysema.

**Case presentation:**

A 49-year-old Thai man with underlying limited cutaneous systemic sclerosis presented with acute low-grade fever, progressive dyspnea and right pleuritic chest pain for five days. Our patient had pulmonary fibrosis with bronchiectasis of both lower lungs related to this underlying disease. He received only low-dose steroid therapy, without any immunosuppressant. A chest radiograph revealed right lung pneumothorax with cloudy yellow color pleural fluid. Cryptococcal pneumonia was diagnosed by positive identification of the cryptococcal antigen in the serum and pleural fluid. His symptoms improved after intercostal drainage and fluconazole therapy.

**Conclusion:**

Infection can exacerbate symptoms in patients with systemic sclerosis with sub-pleural emphysema, thereby triggering a spontaneous pneumothorax. Pleural fluid--present but not initially seen because of the pneumothorax--could be a clue to a pre-existing pulmonary infection.

## Introduction

Systemic sclerosis (SSC) is a rare systemic disease with the classic clinical characteristic of skin tightness. The disease is classified into two major types: limited cutaneous SSC (lcSSC) and diffuse cutaneous SSC (dcSSC). In lcSSC, the skin on the face, neck and below the elbows and knees thickens, while in dcSSC the thickening extends to the trunk, arms and thighs.

Internal organ involvement, particularly pulmonary, is also found in the disease. Pulmonary complications include pulmonary fibrosis, pulmonary arterial hypertension and lung cancer. Most pulmonary fibrosis is found in cases of dcSSC [1]; however, it also occurs in lcSSC [1].

Spontaneous pneumothorax, related to the underlying pulmonary fibrosis--particularly lung cyst or bleb, has been reported as a rare complication of SSC [2,3]. There are, however, no reports of spontaneous pneumothorax in SSC, as related to pulmonary infection. We present a case of underlying lcSSC in a patient who developed spontaneous pneumothorax related to cryptococcal pneumonia.

## Case report

A 49-year-old Thai male was diagnosed with lcSSC according to the 1980 criteria of the American Rheumatism Association [4]. His first presenting symptoms included sclerodactyly, symmetrical polyarthralgia, digital pitting scar and Raynaud's phenomenon without any chest symptoms.

**Figure 1:**
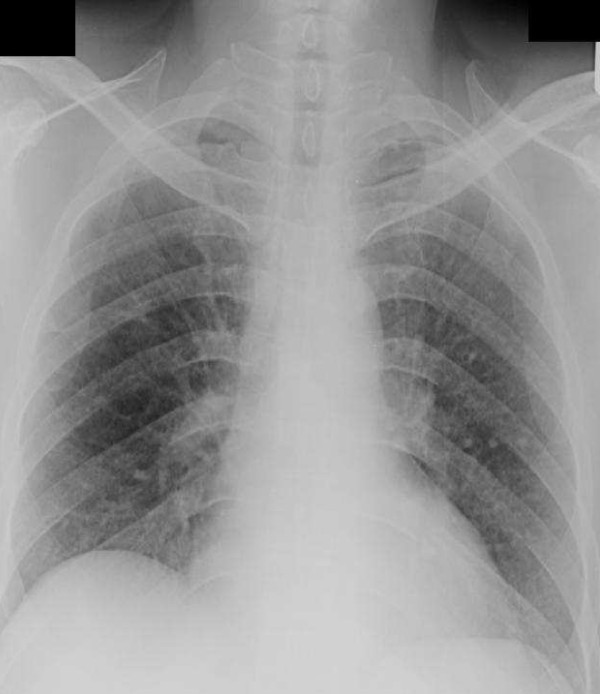
**Chest radiograph showing interstitial infiltration in both lower lung fields**.

**Figure 2:**
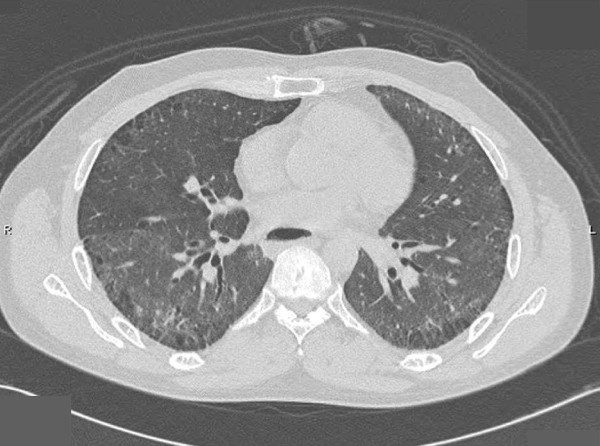
**HRCT chest scan revealed minimal thickening of the interlobular septa with thickening of the pleura in both lungs and mild bronchiectasis in both lower lungs**.

Four years after diagnosis, our patient developed dyspnea on exertion. The dyspnea worsened in the following two years so further investigations were performed. His chest radiograph revealed interstitial infiltration in both lower lung zones and prominent pulmonary trunk (Figure 1). Pulmonary function showed a restrictive pattern; with a forced vital capacity (FVC) of 53%, and forced expiratory volume in one second/forced vital capacity (FEV1/FVC) of 105%. High resolution computer tomography (HRCT) of the chest presented minimal thickening of the interlobular septa with thickening of the pleura and mild bronchiectasis in both lower lungs (Figure 2). An echocardiogram detected mild concentric left ventricular hypertrophy without pulmonary arterial hypertension.

Since our patient's symptoms had worsened, the attending physician prescribed oral daily cyclophosphamide (100 mg per day) plus prednisolone (10 mg per day). Within three months, the clinical symptoms improved; however, the follow-up FVC was minimally decreased with an FVC of 45% and an FEV1/FVC of 90%. Cyclophosphamide was stopped after one year of treatment and the steroid tapered off during follow-up.

**Figure 3:**
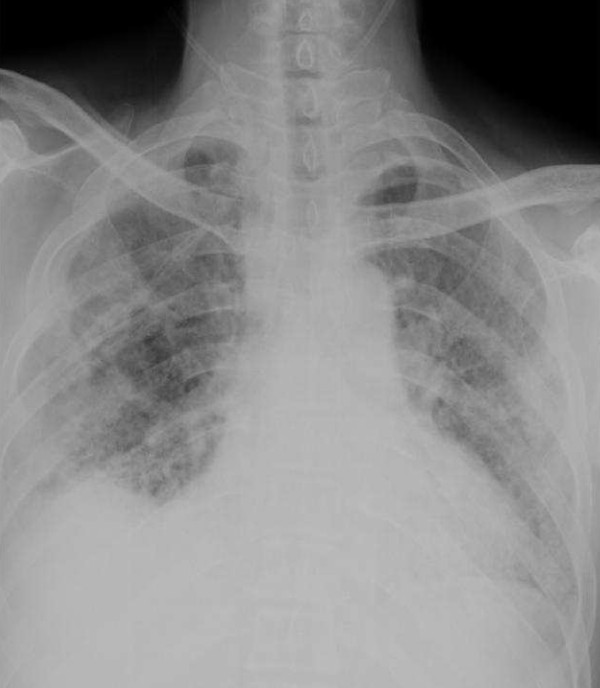
**Chest radiograph showing extensive interstitial infiltration compared to previous chest radiograph**.

**Figure 4:**
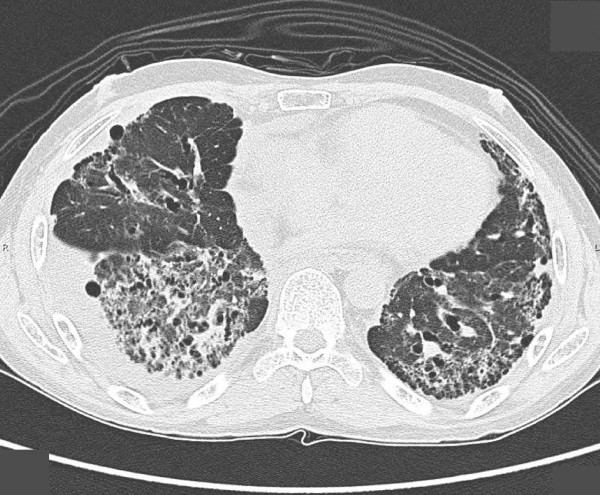
**HRCT chest scan reveals thickening of interlobular septa with sub-pleural emphysema and tubular bronchiectasis of right lower lung**.

One year after stopping the cyclophosphamide--but while still on the prednisolone--the symptoms of dyspnea upon exertion returned. A radiograph and HRCT of the chest were therefore repeated to ascertain the cause of dyspnea. We observed increased thickening of the interlobular septa with sub-pleural emphysema and tubular bronchiectasis of his right lower lung (Figures 3 and 4). Supportive treatment with low-flow oxygen therapy and breathing exercises were prescribed because of the chronic pulmonary fibrosis.

Our patient was admitted because of a low-grade fever with pleuritic chest pain, progressive shortness of breath, and a productive cough of five days duration. On physical examination, he had tachypnea (28 breaths/minute) and a body temperature of 38.2°C. A lung examination revealed decreased breath sounds and vocal resonance of his right lung with a midline trachea position. The oxygen saturation of the room air at admission was 92%.

**Figure 5:**
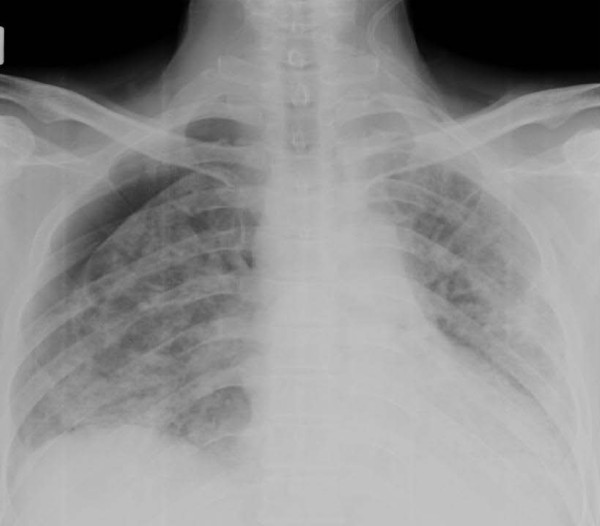
**Chest radiography reveals pneumothorax of right lung**.

A complete blood count indicated a hemoglobin level of 14.2 g/dL, white blood cell count of 8,800 cells/mm^3^, and a platelet count of 247,000 cells/mm^3^. A chest radiograph revealed pneumothorax of his right lung (Figure 5).

Cloudy yellow-colored fluid was found after a chest tube was inserted. The exudative profile of the pleural fluid included red blood cell count 890 cells/mm^3 ^and white blood cell count 1,890 cells/mm^3 ^(polymorphonuclear cells 64%, eosinophils 30% and lymphocytes 6%). Indian ink, Gram stain and acid-fast stain of the pleural fluid and sputum were negative; however, the pleural fluid was positive for cryptococcal antigen. The respective culture of the pleural fluid and serum was positive for *Cryptococcus neoformans*, while the cerebrospinal fluid was negative.

**Figure 6:**
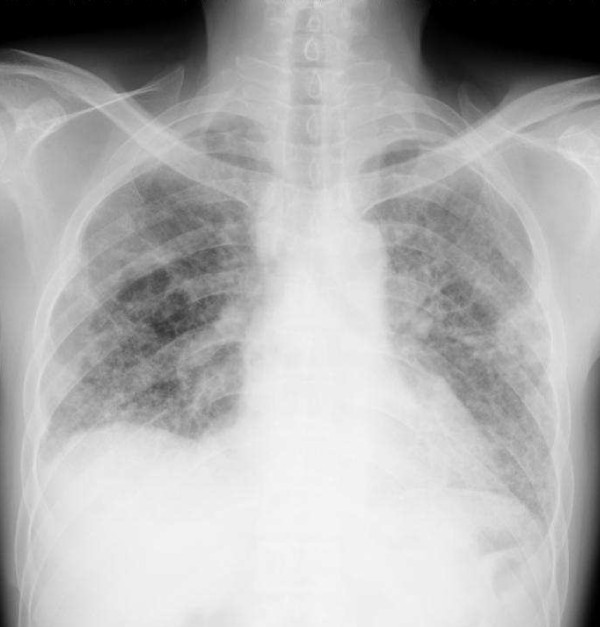
**Chest radiography four weeks after treatment reveals improvement of pneumothorax and pulmonary infiltration**.

Due to desaturation and large air leakage, oxygen supplementation and drainage of his chest were performed immediately. Oral fluconazole (400 mg per day) was prescribed after the presence of Cryptococcus was confirmed (three days after pneumothorax) and continued for six months. The fever, pleuritic chest pain and cough symptoms improved and the lung was re-expanded the third day after chest tube insertion without any complications. The oxygen line was removed seven days after treatment. A chest radiograph indicated improvement of pulmonary infiltration two weeks after treatment (Figure 6). Four weeks after treatment the serum cryptococcal antigen test and hemoculture for *Cryptococci *were negative. No recurrent pneumothorax was detected after anti-fungal therapy was discontinued.

## Discussion

Spontaneous pneumothorax can be a complication of infection from invasive necrotizing organisms such as anaerobic bacteria [5], *Staphylococcus *[6], *Klebsiella pneumoniae *[7], tuberculosis [8,9], aspergillosis [10,11], *Pneumocystis jiroveci *[12], *Scedosporium apiospermum *mycetoma [13] or be associated with pre-existing lung disease such as chronic obstructive pulmonary disease [5], status asthmaticus [5], cystic fibrosis [5], cancer [14], thoracic endometriosis [15] or connective tissue disease [5]. All of the above would be termed secondary spontaneous pneumothorax (SSP).

SSP in rheumatic diseases have been reported in SSC [2,3], polymyositis [16], mixed connective tissue disease [17], systemic lupus erythematosus [18,19], Wegener's granulomatosis [20,21], relapsing polychondritis [22], ankylosing spondylitis [23], and rheumatoid arthritis [24], particularly in persons with a pre-existing lung disease associated with an underlying connective tissue disease. Spontaneous pneumothorax in SSC has been reported in patients with sub-pleural blebs or lung cysts, which are perhaps due to abnormal collagen in the pulmonary tissue as a result of SSC [2,3,25]. An enlarged sub-pleural cyst, particularly > 1 cm, might be a risk for spontaneous pneumothorax in patients with SSC [2].

Our patient had pre-existing pulmonary fibrosis related to his underlying SSC. A HRCT chest scan revealed only sub-pleural emphysema and he developed spontaneous pneumothorax after pulmonary infection. We conclude that, even though sub-pleural blebs occur in SSC in association with spontaneous pneumothorax, infection can exacerbate symptoms in patients with sub-pleural emphysema, thereby triggering spontaneous pneumothorax.

Spontaneous pneumothorax related to cryptococcal pneumonia has been reported, but mostly in underlying acquired immunodeficiency syndrome (AIDS) [26]. There is one report in a healthy young woman [27]. There are no reports of spontaneous pneumothorax related to cryptococcal pneumonia in persons with SSC. The pathophysiology of spontaneous pneumothorax could be a result of lung tissue necrosis due to infection [5], and the pulmonary complications of SSC could be a predisposing factor for pneumothorax in cases where there is a thinning sub-pleural bleb or bullae.

The objectives of treatment in both primary spontaneous pneumothorax (PSP) and SSP are to remove air from the pleural space and to prevent recurrent pneumothorax. Importantly, recurrence is higher and the treatment more difficult in SSP than in PSP [28]; therefore, the management in SSP needs to be more aggressive. However, it is more difficult to make observations without any hospital-based treatment, as often occurs among patients with PSP as opposed to those with SSP, and there is higher morbidity and mortality among the latter [29]. Moreover, because the chest symptoms in SSP are out of proportion with the degree of pneumothorax [30], the intervention must be prompt in those who cannot tolerate pneumothorax even if there is only a low volume of leakage into the pleural space. As for the patient with symptomatic SSP, the treatment should include oxygen supplementation to correct any arterial hypoxemia and air drainage [29]. The treatment options of air drainage in SSP depend on the patient's condition and the size of the air leak into the pleural space.

Our treatment of pneumothorax in daily practice follows The British Thoracic Society (BTS) Pneumothorax Guideline [29]. Accordingly, aspiration can be performed with a 16-18G canula for patients with SSP if the size of the air leakage into pleural space is between 1-2 cm, whereas a chest tube drainage should be inserted if the size is > 2 cm [29]. Our patient presented with severe chest symptoms with hypoxemia and air leakage into pleural space > 2 cm [29], thus there was no doubt about the treatment option. Chest tube drainage and oxygen therapy were immediately performed on our patient. As per the BTS guideline, neither pleurodesis nor surgical strategy was performed on our patient because the lung was re-expanded, the air leak was resolved and there was no recurrence of the pneumothorax (after chest tube drainage).

In most cases, lung re-expansion will lead to rapid recovery; however, re-expansion may be delayed in a patient with SSP [31] or even in SSC [3]. Pneumothorax has been known to recur in cases of SSC [2,25], and so pleurodesis will be the final treatment in most cases of SSC. Slow lung re-expansion and recurrent pneumothorax in SSC may be explained by poor lung compliance, multiple sub-pleural cysts and pleural-thickening and fibrosis [2,3].

In contrast to previous reports, our patient had full lung re-expansion within three days of chest tube insertion and anti-fungal therapy. The rapid recovery of the lung after re-expansion might be related to early anti-fungal treatment and early chest tube drainage. The prognosis of lung re-expansion in SSP due to infection might be better than pneumothorax due to ruptured sub-pleural bleb in a patient with underlying SSC.

In general, pleural effusion will not be detected in the ipsilateral lung of pneumothorax because the pressure in the pleural space will obscure the hydrostatic pressure of the interstitial fluid and there is, therefore, no movement of interstitial fluid into the pleural space [5]. Our patient had pleural fluid after chest tube insertion and the specific cause of pneumothorax came from pleural fluid analysis. Therefore pleural fluid can be a sign of a pre-existing pulmonary infection in cases of spontaneous pneumothorax.

## Conclusions

Infection can exacerbate symptoms in a patient with systemic sclerosis with sub-pleural emphysema, thereby triggering spontaneous pneumothorax, even when minimal sub-pleural emphysema is detected. Pleural fluid--present but not initially seen because of the pneumothorax--could be a clue to a pre-existing pulmonary infection.

## Consent

Written informed consent was obtained from our patient for the publication of this case report and any accompanying images. A copy of the written consent is available for review by the Editor-in-Chief of this journal.

## Competing interests

The authors declare that they have no competing interests.

## Authors' contributions

All authors reviewed and approved the final manuscript.
